# Assessing Outcome of tPA Therapy in Patients with Acute Ischemic Stroke (0-three hours)

**DOI:** 10.12669/pjms.42.8.13372

**Published:** 2026-08

**Authors:** Amna Tariq, Muhammad Adnan Aslam, Qasim Bashir, Sabina Nayab, Aamir Hayat

**Affiliations:** 1 Amna Tariq, FCPS, FEBN. Resident, Department of Neurology, Services Institute of Medical Sciences (SIMS), Lahore, Pakistan; 2 Muhammad Adnan Aslam Head of Department Neurology, Services Institute of Medical Sciences (SIMS), Lahore, Pakistan; 3 Qasim Bashir, MBBS, M.D (USA), ABPN, UCNS (Neurocritical Care), ABMS (Vascular Neurology), FSVIN (USA), CAST Certified in Neuroendovascular Surgery (USA). Head of Department Neurology, Punjab Institute of Neurosciences (PINS), Lahore, Pakistan; 4 Sabina Nayab Resident, Department of Neurology, Services Institute of Medical Sciences (SIMS), Lahore, Pakistan; 5 Aamir Hayat Resident, Department of Neurology, Services Institute of Medical Sciences (SIMS), Lahore, Pakistan

**Keywords:** Acute ischemic stroke, Good functional outcome, tPA

## Abstract

**Objective:**

To determine the frequency of good functional outcome and significant neurological improvement for patients treated with tPA within first three hours of onset of acute ischemic stroke.

**Methodology:**

This cross-sectional study was conducted at Neurology Department, Services Hospital, Lahore, from October 2024 to April 2025. Total 51 patients with acute ischemic stroke who received tPA within three hours of symptom onset were included. Neurological improvement was assessed using NIHSS, and functional outcome with Modified Rankin Scale at baseline and after 90 days; ≥4-point improvement in NIHSS was considered as significant neurological improvement, while mRS scores of 0-2 indicated good functional outcome. Data were analyzed using SPSS v26.

**Results:**

Mean NIHSS score improved from 12.31 ± 5.42 at baseline to 7.96 ± 7.05 at 90 days (p < 0.001), while mean mRS improved from 4.02 ± 1.04 to 2.90 ± 1.81 (p < 0.001). Significant neurological improvement was seen in 54.9% of patients, and 49.0% achieved good functional outcomes. No statistically significant associations were found between gender, age, time of tPA administration, or stroke subtype and outcomes (p > 0.05).

**Conclusion:**

These findings suggest that while intravenous tPA therapy led to meaningful neurological and functional improvement in many patients, baseline demographics and time to tPA administration within the three hours window did not significantly influence the likelihood of achieving favourable outcomes.

## INTRODUCTION

Stroke, remains a leading medical emergency worldwide.^1^ In 2015, Global data reported that 11% of all deaths were attributed to acute stroke, highlighting its substantial impact on public health.^2^ Among strokes, ischemic strokes account for 80% cases, and early emergency management plays crucial role in improving clinical outcomes in these patients.^3^

The 2018 guidelines from American Stroke Association emphasize importance of prompt intervention in acute ischemic stroke, reinforcing that early administration of thrombolytic therapy significantly enhances recovery.^4^ In this context, use of tPA remains the only FDA-approved pharmacological treatment. Its therapeutic advantage is taken when administered within 4.5 hours.^5^ However, delayed administration is associated with increased risk intracranial haemorrhage and reperfusion injury.^6^ Reperfusion injury occurs due to disruption of blood-brain barrier, which is further exacerbated by delayed vessel recanalization. This can lead to cerebral edema, hemorrhagic transformation, and ICH.^7^ Despite the development of various neuroprotective agents aimed at counteracting ischemic damage, most have failed to demonstrate clinical efficacy, even when administered early in stroke timeline.^8^

The ECASS III trial defined favorable outcomes as Modified Rankin Scale score of 0-1 and unfavorable outcomes as scores of 2-6. Multiple studies have supported the use of tPA in improving functional outcomes when administered within recommended time frame.^9^ Baseline NIHSS scores have also been shown to be strong predictors of long-term functional outcomes. Studies suggest that early improvement within first 24 hours and lower baseline NIHSS scores are associated with better prognosis and reduced long-term mortality.^10^

Although the efficacy of intravenous tPA within 3–4.5 hours of ischemic stroke onset is well established internationally, there is a paucity of local data regarding its real-world effectiveness in Pakistan. Differences in healthcare infrastructure, patient characteristics, and delays in presentation may influence treatment outcomes. This study aimed to evaluate functional and neurological outcomes following tPA administration in a tertiary care public hospital and provide local evidence to guide stroke management in our population.

## METHODOLOGY

This descriptive cross-sectional study was conducted at Neurology Department, Services Hospital, Lahore, from October 2024 to April 2025.

### Ethical statement:

The study was permitted by ethical committee of Services Hospital, Lahore [Ref No: IRB/2024/1433/SIMS Dated: 14/09/2024]. Written informed consent was obtained from participants before enrollment. All procedures were carried out in line with ethical principles outlined in Declaration of Helsinki and its later revisions or equivalent ethical standards.

### Study population:

Sample size of 51 patients was calculated using WHO sample size calculator, with expected proportion of 34% for good functional outcome after three months of tPA administration,^11^ margin of error 11%, and 90% confidence level. Following formula used;


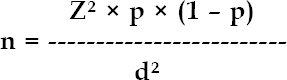


Where, Z = 1.645 (for 90% confidence level), p = 0.34 (34% good functional outcome) q = 1 – p = 0.66 and d = 0.11 (11% margin of error).

For patients enrollment non-probability consecutive sampling technique was used.

Patients aged between 18 and 80 years, with diagnosis of ischemic stroke and measurable neurologic deficit based on NIHSS, and who received tPA within three hours of symptom onset with no hemorrhage on initial CT brain were included in the study. To ensure homogeneity and accuracy, following exclusion criteria were applied: stroke or head trauma within three months, major surgery (two weeks), prior intracranial hemorrhage, BP>185/110 mmHg, rapidly improving symptoms, GIT or urinary tract bleeding (within three weeks), arterial puncture (within seven days), use of anticoagulants within 48 hours, INR ≥1.7, platelet count <105/μL, or BSL <50 or >400 mg/dL. Patients with MI within past three months, or those who lost to follow up were also excluded.

### Study objective:

The objective was to determine the frequency of patients achieving good functional outcome and significant neurological improvement 90 days following intravenous tPA therapy.

### Data collection:

For data collection pre-designed proforma was used. Clinical data were collected at time of admission and during follow-up. NIHSS scores and Modified Rankin Scale (mRS) were recorded: before tPA administration, and at 90 days. All patients received standard tPA dose of 0.9 mg/kg (maximum 90 mg), with 10% as initial bolus and remainder infused over one hour. Functional outcome was assessed using mRS at 90 days post-treatment, patients with 0-2 score was labelled as having good functional outcome - GFO. A significant neurological improvement was defined as improvement of ≥4 points on NIHSS. Collected data included patient demographics (age, gender), medical history, tPA administration time, baseline and follow-up mRS, and NIHSS scores. Patients who lost to follow up or expired during study duration were excluded to maintain integrity of data.

### Statistical analysis:

Data analyzed using SPSS version 26. Numerical variables were expressed as frequencies and percentages, while continuous as mean ± standard deviation. Data was stratified for effect modifiers to find out the effect of these on outcomes, through chi square. The mean differences in NIHSS and mRS scores were analyzed using paired samples t-test, with p-value of less than 0.05 regarded as statistically significant.

## RESULTS

A total of 51 patients who met the inclusion criteria were enrolled in this study. The majority of the participants were male (71%), with females accounting 21%. Most patients (76.5%) were over the age of 50, while only 23.5% were aged 50 or below. Regarding medical history, 56.9% had diabetes, 62.7% had hypertension, 33.3% had history of IHD. and 9.8% had previous stroke. The time from symptom onset to tPA administration varied, with 27.4% receiving within first hour, 39.2% within 60-120 minutes, and 33.4% between 120-180 minutes. In terms of stroke subtype, large vessel occlusion was most common (68.6%), followed by small vessel occlusion (17.6%), cardiac embolism (11.8%), and others (2.0%).

A statistically significant improvement was observed in both NIHSS and mRS scores at 3-month follow-up. The mean NIHSS score decreased from 12.31 ± 5.42 at baseline to 7.96 ± 7.05 (p < 0.001), and mean mRS score improved from 4.02 ± 1.04 to 2.90 ± 1.81 (p < 0.001), indicating both significant neurological recovery and functional improvement in study cohort (Table-I). Assessment of outcomes revealed that 28 patients (54.9%) achieved significant neurological improvement (≥4-point reduction in NIHSS), while 25 patients (49.0%) achieved good functional outcome (mRS score 0-2) at 90 days post-tPA administration.

**Table-I:** Outcomes and Mean NIHSS and MRS scores before and after three months follow up (N=51).

Descriptive	Before treatment	At three months	p-value
NIHSS score	12.31±5.42	7.96±7.05	<0.001*
MRS score	4.02±1.04	2.90±1.81	<0.001*
Significant Neurological improvement	28 (54.9%)		
GFO	25 (49.0%)		

Chi-square analysis showed no statistically significant association between neurological improvement or good functional outcome and gender (p = 0.563 and 0.691 respectively), age group (p = 0.785 and 0.460), or tPA administration time (p = 0.434 and 0.769). While small vessel occlusion showed trend toward better outcomes (78% achieving neurological improvement and 77.8% achieving GFO), association between stroke subtype and outcomes was not statistically significant (p = 0.259 and 0.162) (Table-II).

**Table-II:** Chi square test to see association between different variables and outcomes.

Variables	Categories	Significant Neurological improvement		GFO		p-value	
		NIHSS score		MRS Score			
		Yes	No	Yes	No	NIHSS	MRS
		N (%)	N (%)	N (%)	N (%)		
Gender	Female	8(53)	7(47)	8(53)	7(47)	0.563	0.691
	Male	20(56)	16(44)	17(47)	19(53)		
Age	Upto 50 years	7(58)	5(42)	7(58)	5(42)	0.785	0.460
	above 50 years	21(54)	18(46)	18(46)	21(54)		
Time of tPA	1 hour	6(42)	8(58)	6(42)	8(58)	0.434	0.769
	2 hours	13(65)	7(35)	11(55)	9 (45)		
	three hours	9(53)	8(47)	8(47)	9(53)		
Stroke type	Small vessel occlusion	7(78)	2(22)	7(77.8)	2(22.2)	0.259	0.162
	Large vessel occlusion	18(52)	17(48)	15(40)	20(60)		
	Cardiac emboli	2(33)	4(67)	2(33)	4(67)		
	Others	1(100)	0(0)	1(100)	0(0)		

## DISCUSSION

The majority of our patients were male and older than 50 years, and hypertension and diabetes were the most common comorbidities, findings that are in agreement with established epidemiological patterns of ischemic stroke.^12^,^13^ Libruder et al. similarly reported hypertension (64.9%) and diabetes (25.7%) as the most prevalent vascular risk factors among ischemic stroke patients.^14^ A substantial proportion of our cohort had large vessel occlusion, which is also consistent with contemporary literature demonstrating the significant burden of this stroke subtype.^15^,^16^

The present study demonstrated significant neurological and functional improvement following intravenous tPA administration in acute ischemic stroke patients. The mean NIHSS score significantly decreased from 12.31±5.42 at baseline to 7.96±7.05 at three months (p<0.001), while the mean mRS score improved from 4.02±1.04 to 2.90±1.81 (p<0.001). Significant neurological improvement was observed in 54.9% of patients, and good functional outcome (mRS 0–2) at three months was achieved in 49% of patients. The proportion of patients achieving good functional outcome in our study is comparable to that reported by Marko et al., who demonstrated favorable functional outcomes ranging from 45.9% to 56.8% following intravenous thrombolysis. Their multivariable analysis further showed that increasing age (OR 0.34 for age 71–80 years), female sex (OR 0.84), and higher baseline NIHSS score (OR 0.86) were associated with lower odds of favorable outcomes.^17^ Likewise, Palaiodimou et al., in pooled analysis of 11 trials involving 3,757 patients treated with intravenous tPA, reported good functional outcomes in 46%–50% of patients at three months and significantly reduced disability (p=0.034), findings that closely mirror our results.^18^ In contrast, Wang et al. reported substantially higher frequency of good functional outcome (71.3%) following thrombolysis, although they did not observe significant improvement in NIHSS score (p=0.194).^19^ The superior outcomes observed in their study may be explained by differences in baseline stroke severity, younger patient population, earlier presentation, and better access to organized stroke care and rehabilitation services.

In the present study, no statistically significant associations were observed between functional outcomes and age, gender, stroke subtype, or time to tPA administration. Conversely, Marko et al, & Wang et al, also demonstrated better outcomes among younger patients, although they similarly found no gender-based differences.^17^,^19^ Furthermore, Alaka et al. identified older age, higher baseline NIHSS score, and uncontrolled hypertension as independent predictors of poor functional outcome.^20^ The lack of significant predictors in our study may be attributable to the modest sample size and limited statistical power to detect such associations.

Our findings are also comparable to local data from Pakistan. A study conducted in Karachi among 79 ischemic stroke patients treated with low-dose tPA reported functional independence at three months in 50.6% of patients receiving low-dose therapy and 54.8% of those receiving standard-dose therapy, figures remarkably similar to our observed good functional outcome of 49%.^21^ However, unlike that study, we did not compare different tPA dosing regimens or assess symptomatic intracranial hemorrhage.

### Strength:

A major strength of the present study is that it provides real-world data on the effectiveness of intravenous thrombolysis from a tertiary care public hospital in Pakistan, where local evidence remains scarce. Nevertheless, larger multicenter prospective studies with long-term follow-up and systematic assessment of post-thrombolysis complications are needed to better identify predictors of outcome and further evaluate the safety and effectiveness of tPA in our population.

### Limitations:

This study has several limitations. Important safety outcomes, including in-hospital mortality and symptomatic intracranial hemorrhage, were not monitored or recorded during the hospital stay. Consequently, study primarily assessed functional outcomes and could not comprehensively evaluate the safety profile of intravenous tPA in our population. The absence of complication monitoring may have introduced information bias and created gap between the observed functional outcomes and true overall impact of thrombolytic therapy. Future prospective studies incorporating systematic in-hospital monitoring of adverse events and mortality are required to provide more complete assessment of the effectiveness and safety of tPA in our setting.

## CONCLUSION

These findings suggest that while intravenous tPA therapy led to meaningful neurological and functional improvement in many patients, baseline demographics and time to tPA administration within the three-hour window did not significantly influence the likelihood of achieving favourable outcomes.

### Authors Contribution:

**AT:** Conceived and designed the study, data collection, manuscript drafting.

**MAA:** Supervision, critical review, editing, and final approval of manuscript.

**QB:** Technical guidance, interventional neurology input, departmental oversight, review, editing, accountable for the accuracy of the work.

**SN, AH:** Assisted in data collection, analysis, and manuscript writing.
